# Matrix-Bound and Media-Derived Extracellular Vesicles from Mineralized Osteoblasts Exhibit Distinct Osteogenic Activities

**DOI:** 10.3390/ijms27167400

**Published:** 2026-08-19

**Authors:** Julien Guerrero, Chafik Ghayor, Ana Pérez Domínguez, Indranil Bhattacharya, Franz E. Weber

**Affiliations:** 1 Center of Dental Medicine, Oral Biotechnology & Bioengineering, University of Zurich, 8032 Zurich, Switzerland; julien.guerrero@usz.ch (J.G.); chafik.ghayor@usz.ch (C.G.); ana.perez@usz.ch (A.P.D.); indranil.bhattacharya@usz.ch (I.B.); 2 CABMM—Center for Applied Biotechnology and Molecular Medicine, University of Zurich, 8057 Zurich, Switzerland

**Keywords:** extracellular vesicles, matrix-bound extracellular vesicles, media-derived extracellular vesicles, osteoblasts, osteogenesis, mesenchymal stromal cells, extracellular matrix, bone regeneration, RNA cargo, bone tissue engineering

## Abstract

Extracellular vesicles (EVs) derived from osteoblasts are emerging as key regulators of bone formation, yet functional differences between vesicles from distinct extracellular compartments remain unclear. In this study, we compared media-derived extracellular vesicles (MEs) and matrix-bound extracellular vesicle-enriched fractions (MBEs) isolated from mineralized osteoblasts (MOBs) and evaluated their effects on human bone marrow-derived mesenchymal stromal cells (hBMSCs). Both preparations, characterized by nanoparticle tracking analysis and transmission electron microscopy, displayed similar size distributions (30–200 nm) and concentrations. Moreover, both preparations showed enrichment of the EV-associated marker CD63, with no detectable GAPDH and only minimal Grp94 signals in a subset of samples. mRNA profiling revealed that MBE-enriched fractions were selectively enriched in RUNX2, whereas other osteogenesis-related transcripts were comparable between them. Functional analyses demonstrated that both EV populations promoted osteogenic differentiation of hBMSCs, although with distinct biological profiles. MBE-enriched fractions were associated with higher alkaline phosphatase activity under control conditions, whereas MEs induced greater osteocalcin expression and showed a numerical tendency toward increased matrix mineralization, particularly under osteogenic conditions. These findings suggest that extracellular vesicles associated with different extracellular compartments exhibit distinct osteogenic activities rather than a uniform biological effect. Although the matrix-bound preparation likely contains extracellular matrix-associated components co-isolated during the extraction procedure, the present study highlights the importance of extracellular compartmentalization in shaping EV-associated bioactivity and provides a foundation for future studies aimed at optimizing EV-based strategies for bone regeneration.

## 1. Introduction

Bone is a dynamic tissue whose structure and function are continuously remodeled by coordinated actions of osteoblasts, osteoclasts and osteocytes [1,2]. This homeostatic remodeling is critical for fracture repair, metabolic mineral balance, and adaptation to mechanical load [3,4]. Failure of these processes underlies fragility fractures and delayed bone repair, which represent significant clinical burdens worldwide. Modern regenerative strategies therefore seek biologically potent, translationally feasible approaches to stimulate osteogenesis, while avoiding the drawbacks of cell transplantation or high-dose protein therapies [5].

Extracellular vesicles (EVs), a heterogeneous collection of membrane-bound particles (including exosomes, microvesicles, apoptotic bodies, and matrix-associated vesicles), have emerged as central mediators of intercellular communication in development, homeostasis and regeneration [6,7]. Extracellular vesicles are commonly defined as vesicles from 50 to 200 nm, of endosomal origin, released after multivesicular body (MVB) fusion with the plasma membrane [8,9]. They carry proteins, lipids, mRNAs and regulatory non-coding RNAs that can modulate recipient cell behavior. Microvesicles and classical matrix vesicles are typically plasma-membrane-derived and may show overlapping size ranges and cargos, which complicates taxonomic distinctions. Importantly, EVs not only act as mobile paracrine effectors distributed throughout tissue fluids, but a subset of EVs become physically associated with or trapped within the extracellular matrix (ECM). Such EVs are often referred to as matrix-bound extracellular vesicles (MBEs), and they appear to constitute an integral bioactive component of the ECM [10,11,12].

Historically, MBEs were first described as mineral nucleation centers produced by mineralizing cells (osteoblasts, chondrocytes and odontoblasts) [13,14]. They concentrate alkaline phosphatase (TNAP), annexins and phosphatidylserine, create a microenvironment favorable to apatite nucleation, and thus play a direct physicochemical role in the earliest steps of biological mineralization [14,15,16]. Advances in imaging, proteomics and lipidomics have recently expanded our conception of matrix-associated vesicles to include a class of MBEs that possess distinct lipid, RNA and protein signatures and which appear to act as ECM-anchored signaling organelles that influence local cell behavior (for example immune modulation and support of tissue repair) [17,18,19]. These discoveries have blurred earlier categorical boundaries and prompted renewed interest in discriminating vesicle subtypes by origin, cargo and function [20,21].

At the intersection of bone biology and EV research, a growing body of studies shows that osteoblast-derived EVs collected from conditioned media (here called MEs) can transfer osteoinductive cargoes that enhance osteogenic differentiation of mesenchymal stromal cells (MSCs) in vitro and promote bone formation in vivo [22,23,24]. Proteomic and functional analyses implicate EV-associated annexins, collagens and non-collagenous matrix proteins as contributors to nucleation and matrix assembly, and multiple groups have found that EVs derived from mineralizing osteoblasts are more osteoinductive than EVs from non-mineralizing cells [25,26,27]. Crucially, the biological effect of these media-released EVs is influenced by the osteogenic status of the parent cell and by the microenvironment in which EVs are applied (e.g., requirement for phosphate supply to reveal mineralizing activity) [5].

Concurrently, MBEs that are recovered from decellularized/mineralized ECMs have been shown to differ from media EVs in composition and biological actions [17,18]. MBEs often lack canonical extracellular vesicle markers (CD9, CD63, CD81, and HSP70), display tissue-specific protein and lipid signatures, and convey ECM-dependent signals that modulate cell proliferation, immune responses and repair processes in a manner that reflects their matrix niche [18,19]. Because MBEs are physically anchored to the ECM in vivo, they represent a distinct reservoir of stable, matrix-tethered bioactivity that may act locally to regulate progenitor cell behavior during repair and remodeling [17,18]. Such differences in origin and molecular cargo raise the fundamental possibility that MEs and MBEs (or MBVs) are not functionally redundant and may engage recipient marrow stromal cells through separate molecular mechanisms [10,11].

From the mechanistic perspective, several features differentiate matrix-associated vesicles from freely secreted media EVs and suggest distinct functional consequences for human bone marrow mesenchymal stromal cells (hBMSCs). First, MBEs are relatively enriched in mineralization-related proteins (e.g., tissue-nonspecific alkaline phosphatase, annexins) and in phospholipids such as phosphatidylserine that favor calcium binding and nucleation. Second, MBEs may be enriched in specific miRNA and protein cargoes that reflect long-term ECM programming and could modulate MSC lineage commitment, immune signaling and matrix remodeling in a context-dependent way [28]. Third, vesicle uptake pathways and kinetics may differ depending on surface ligands and the physical state (matrix-bound vs. soluble), influencing the intracellular routing and bioavailability of cargo. Collectively, these distinctions imply that MBEs might preferentially prime resident MSCs for matrix remodeling and local osteoinduction, whereas MEs could act more broadly as paracrine messengers that stimulate osteogenic programs via different receptor or endocytic pathways [10,20].

Several translational and conceptual motivations justify a direct, side-by-side comparison of MEs and MBEs on hBMSCs. EV-based, cell-free therapeutics are attractive because they avoid some risks and logistic issues inherent to cell therapy, and early preclinical studies suggest osteoblast-derived EVs can approximate or even exceed BMP-2 in certain in vitro assays of mineralization when supplied under appropriate conditions [22,24,29]. Conversely, MBEs, due to their ECM association, may be more easily incorporated into biomaterials and scaffolds as a stable, matrix-anchored osteoinductive signal [24,27,30]. However, the literature remains fragmented: isolation protocols vary (differential centrifugation and density gradients for media EVs; decellularization and matrix digestion/extraction approaches for MBEs), characterization criteria are inconsistent, and functional comparisons using matched parent cell sources and standardized dosing on primary human BMSCs are scarce [6,31,32]. These methodological and conceptual gaps hinder rational development of EV/MBV-based bone therapies and limit our understanding of how the ECM niche shapes vesicular signaling during bone formation [5,10].

Accordingly, we designed a comparative study to isolate and characterize two operationally defined EV-enriched preparations produced by the same mineralizing osteoblasts: (1) MEs obtained from conditioned culture media during mineralizing culture, and (2) MBEs recovered from the mineralized extracellular matrix produced by those osteoblasts. We systematically compare size distribution, canonical and matrix-associated protein markers, and nucleic acid cargo (selected mRNAs known to regulate osteogenesis). Functionally, we evaluate effects of EVs on hBMSC proliferation, a marker associated with early osteogenic differentiation (e.g., RUNX2, ALP), and matrix mineral deposition, readouts relevant to bone healing. This design tests the hypothesis that MBE-enriched fractions and MEs, despite a shared cellular origin, possess distinct molecular signatures and differential potency/mechanisms of action on hBMSCs, with implications for EV-based biomaterials and cell-free bone regeneration strategies [5,20].

In summary, current evidence positions both media-released and matrix-bound extracellular vesicles from osteoblasts as promising mediators of osteogenic signaling, but direct comparative data on how matrix-anchored versus freely secreted vesicles influence human marrow stromal progenitors are limited. Resolving this question will clarify basic mechanisms of vesicle-mediated bone formation, improve ME and MBE isolation and characterization standards, and inform translational decisions about whether cell-free bone repair strategies should prioritize soluble EV delivery, matrix-incorporated vesicles, or hybrid approaches. The present study aims to fill this gap by providing a rigorous molecular and functional comparison of MEs and MBE-enriched fractions derived from mineralizing osteoblasts on primary human BMSCs [21].

## 2. Results

**Figure 1:**
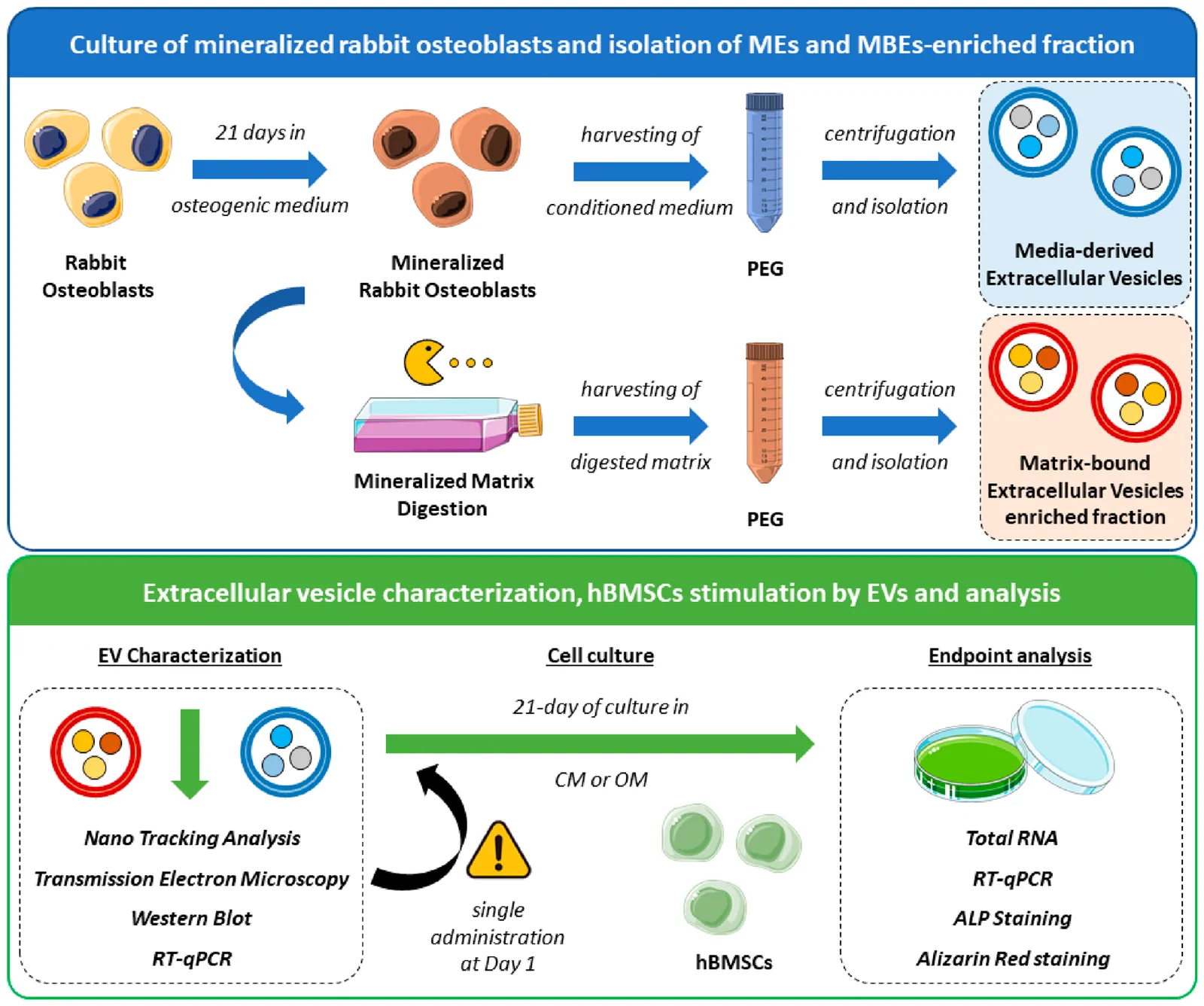
Experimental design and workflow of the study. Primary rabbit osteoblasts (rOBS) were cultured for 21 days under mineralizing conditions to generate mineralized osteoblast cultures. Two operationally defined extracellular vesicle (EV) populations were subsequently isolated from the same cultures: media-derived extracellular vesicles (MEs), recovered from conditioned medium collected during the final 48 h of culture in EV-depleted medium, and a matrix-bound extracellular vesicle-enriched fraction (MBE), recovered from the mineralized extracellular matrix following collagenase digestion. Both EV preparations were characterized by nanoparticle tracking analysis (NTA) to determine particle size distribution and concentration, transmission electron microscopy (TEM) to assess vesicular morphology, Western blotting for EV-associated and intracellular marker proteins, and RT-qPCR to analyze selected EV-associated mRNAs. Equal particle numbers of MEs or the MBE-enriched fraction were then administered once to human bone marrow-derived mesenchymal stromal cells (hBMSCs) on Day 1, followed by culture for 21 days in either complete medium (CM) or osteogenic medium (OM). On Day 21, the biological effects of the EV preparations were evaluated by total RNA isolation, RT-qPCR analysis of osteogenic gene expression, alkaline phosphatase (ALP) staining, and Alizarin Red S staining to assess matrix mineralization. The schematic summarizes the timing of EV isolation, characterization, treatment, and endpoint analyses performed in the study.

To investigate the osteogenic potential of MEs and MBE-enriched fractions isolated from MOBs on hBMSCs, we first characterized and compared both vesicle populations derived from the same cellular source (Figure 1).

### 2.1. Biophysical Characterization of MEs and MBE-Enriched Fractions

**Figure 2:**
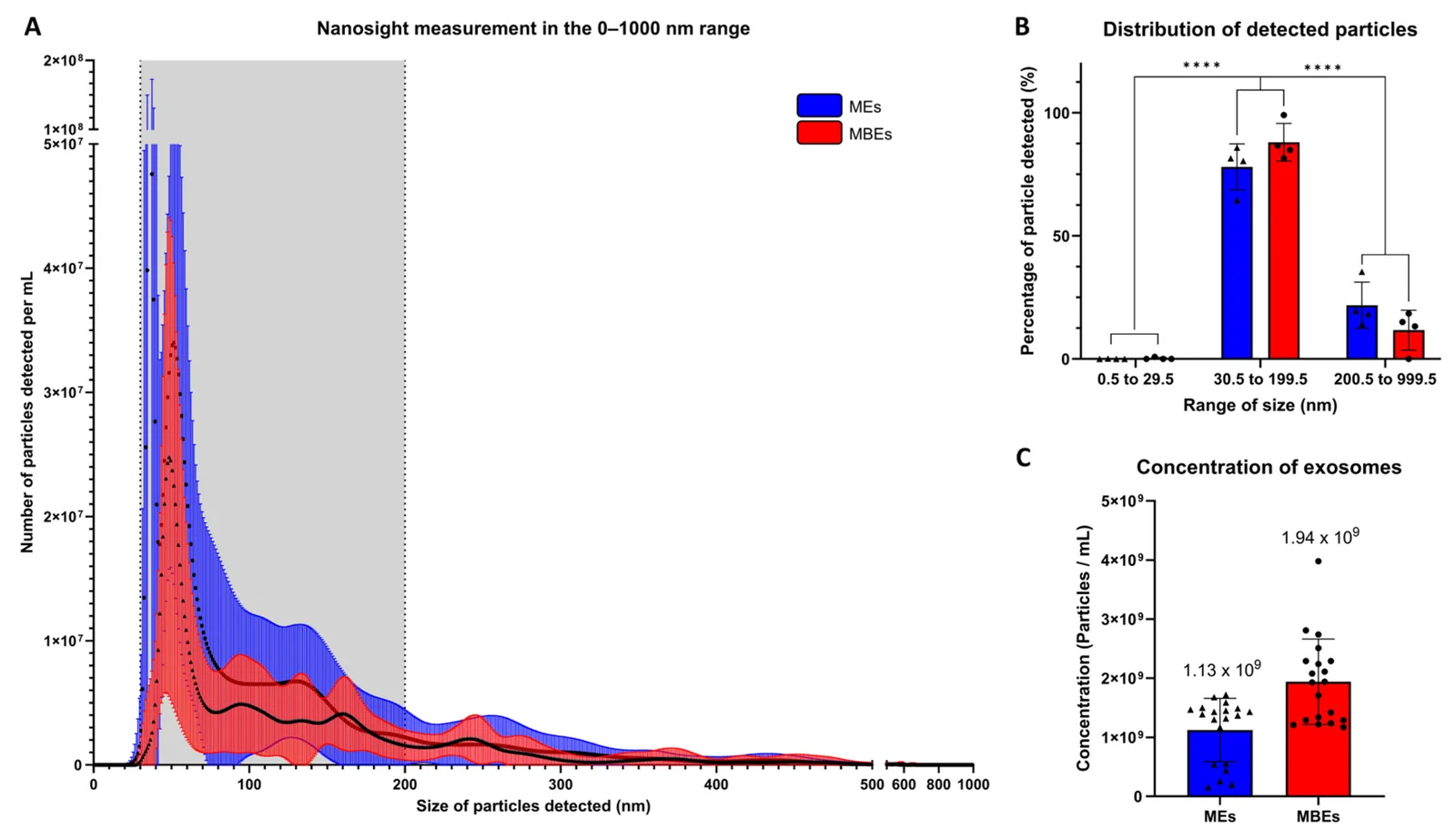
Nanoparticle tracking analysis (NTA) of extracellular vesicles isolated from mineralizing osteoblasts. (**A**) Size distribution profiles of media-derived extracellular vesicles (MEs) and matrix-bound extracellular vesicle-enriched fractions (MBEs). (**B**) Proportion of particles within defined size ranges, highlighting the percentage of vesicles within the extracellular vesicle size range (30–200 nm). (**C**) Particle concentration of MEs and MBE-enriched fractions following isolation. Data are presented as mean ± SD. Statistical significance is indicated as **** *p* < 0.0001.

The size distribution and concentration of MEs and MBE-enriched fractions were assessed using nanoparticle tracking analysis (NTA) (Figure 2). Both vesicle populations displayed comparable size profiles, with no significant differences between conditions. A minor particle peak around 36 nm was observed in ME samples (Figure 2A), but this did not affect the overall distribution. In both conditions, the majority of detected particles exhibited diameters between 30 and 200 nm, accounting for 83.1 ± 9.3% and 88.1 ± 7.7% of total detected particles in ME and MBE-enriched fraction preparations, respectively (Figure 2B).

Quantification revealed similar vesicle concentrations between groups, with 1.13 × 10^9^ ± 5.35 × 10^8^ particles/mL for MEs and 1.94 × 10^9^ ± 7.23 × 10^8^ particles/mL for MBE-enriched fractions (Figure 2C). These results indicate that MEs and MBE-enriched fractions exhibit comparable biophysical properties in terms of size distribution and abundance, supporting their suitability for subsequent functional comparisons.

### 2.2. Extracellular Vesicle Markers Within MEs and MBE-Enriched Fractions

**Figure 3:**
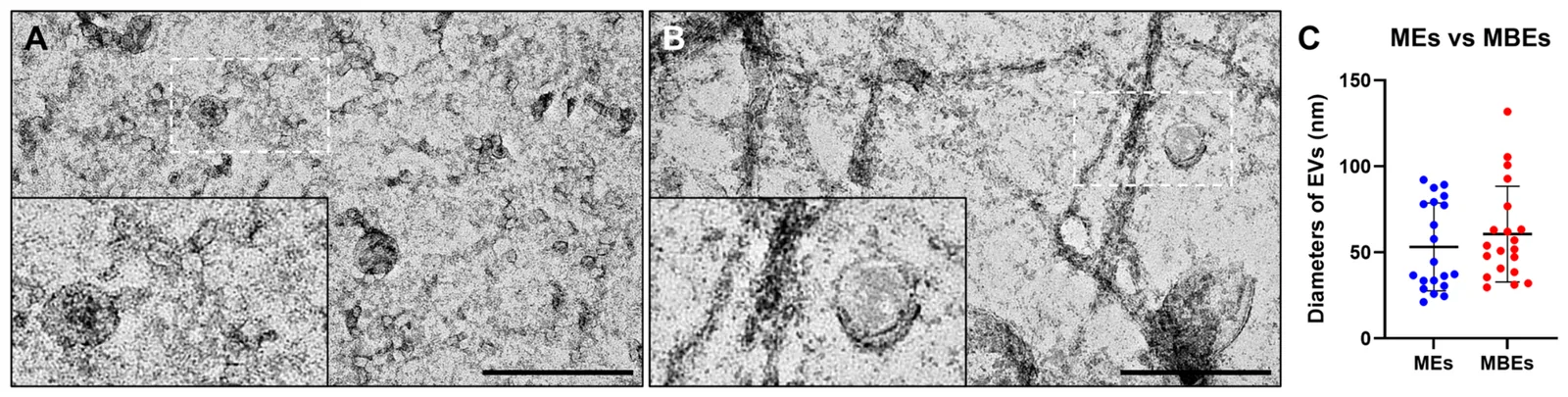
Characterization of EVs derived from mineralized osteoblasts with TEM. (**A**) MEs derived from mineralized OBs. (**B**) MBE-enriched fractions derived from mineralized OBs. In (**A**,**B**), the white dashed box indicates the region selected for magnification, and the solid black box outlines the corresponding magnified inset shown in the bottom-left corner. The scale bar represents 300 nm. (**C**) Measurement of the diameters of MEs and MBE-enriched fractions.

Negative-staining transmission electron microscopy (TEM) demonstrated vesicle-like structures in both preparations of MEs and MBE-enriched fractions (Figure 3). While MEs consisted predominantly of isolated vesicular structures (Figure 3A), MBE-enriched fraction preparations additionally contained irregular fragments, consistent with residual extracellular matrix material (matrix-associated material) originating from the mineralized osteoblast cultures (Figure 3B). Notably, the EVs appeared spatially separated from these fragments rather than being directly attached to their surface, indicating that the vesicles remained freely suspended following the isolation procedure. Additionally, in both preparations, the vesicles exhibited the characteristic rounded morphology of extracellular vesicles (EVs). The diameters ranged from 20 to 100 nm for MEs (mean ± SD: 53.18 ± 25.44 nm) and from 30 to 130 nm for MBE-enriched fractions (mean ± SD: 60.64 ± 27.82 nm) (Figure 3C).

**Figure 4:**
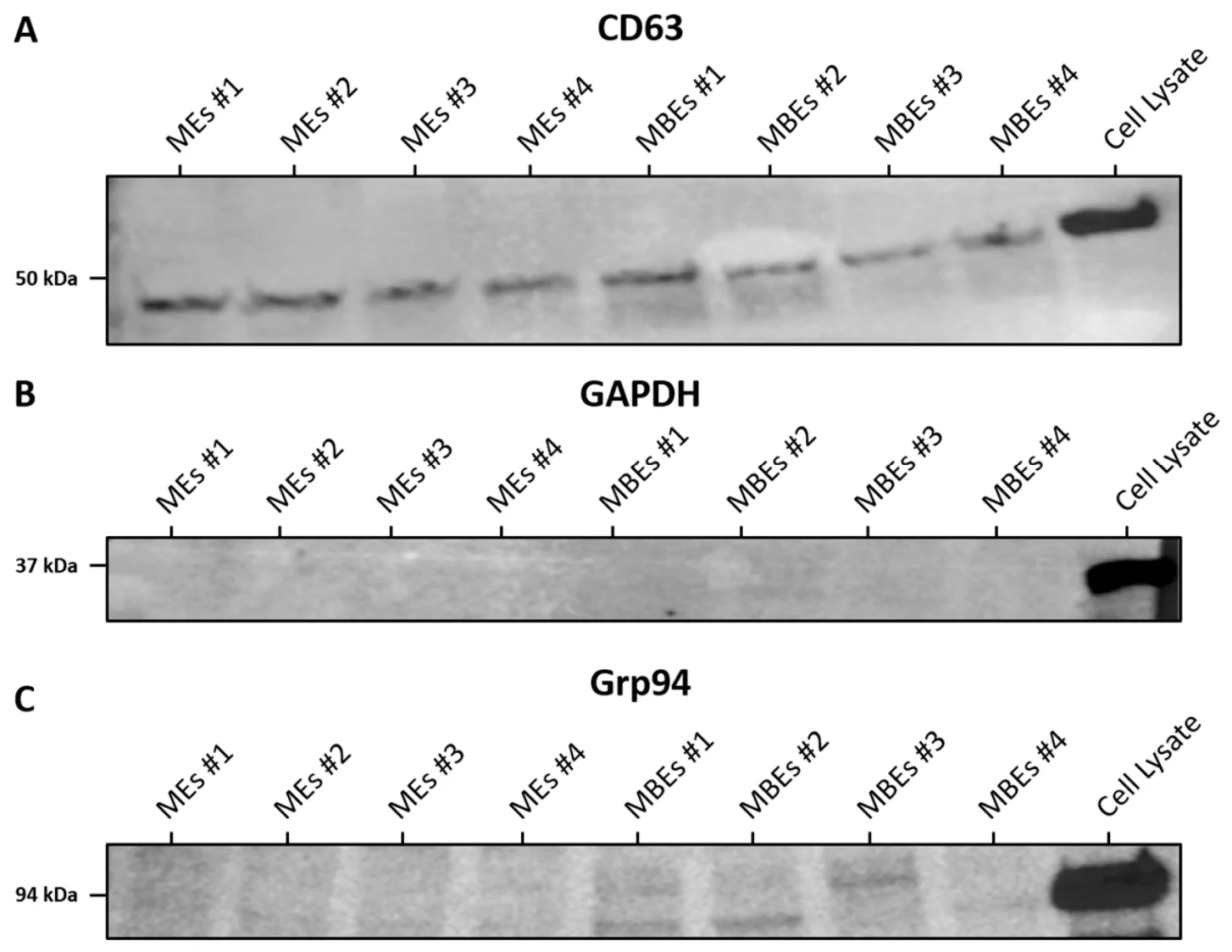
Western blot characterization of media-derived extracellular vesicles (MEs), matrix-bound extracellular vesicle-enriched fractions (MBEs), and cell lysates from mineralized osteoblasts (MOBs). Four independent samples were analyzed for each EV population (samples #1–#4). Immunoblotting was performed for (**A**) the EV-associated marker CD63, (**B**) the cytosolic marker GAPDH, and (**C**) the endoplasmic reticulum-associated protein Grp94.

To further validate the identity and purity of MEs and MBE-enriched fractions isolated from MOBs, we performed Western blot analyses using protein markers recommended by the MISEV guidelines (Figure 4 and Figure S1) [6]. Specifically, we assessed the presence of the tetraspanin CD63 (Category 1, transmembrane or GPI-anchored proteins enriched in EVs), the cytosolic protein GAPDH (Category 2), and the endoplasmic reticulum-associated chaperone Grp94 (Category 4, negative marker for EV preparations), as previously described [33].

Western blot analysis revealed clear enrichment of CD63 in both ME and MBE-enriched fractions isolated from MOBs, supporting the presence of extracellular vesicles (Figure 4A). This enrichment was consistently observed in all four ME samples and all four MBE-enriched fraction samples analyzed (donors #1–#4). CD63 signal was also detectable in MOB cell lysates, in agreement with the known intracellular localization of tetraspanins within endosomal and multivesicular body compartments prior to vesicle secretion. In contrast, GAPDH was readily detected in MOB cell lysates but was not detected from both ME and MBE-enriched fraction preparations, with no detectable signal in any of the four ME or MBE-enriched fraction samples analyzed, indicating minimal cytosolic contamination of the isolated vesicles (Figure 4B). Grp94 was strongly detected in cell lysates but was detected only as faint bands in a subset of EV samples, specifically in ME donors #3 and #4 and in MBE-enriched fraction donors #1 and #2, indicating effective exclusion of intracellular secretory pathway contaminants and supporting the overall purity of the vesicle preparations (Figure 4C). Upon close inspection, very faint bands may be observed in both extracellular vesicle fractions; however, these signals are negligible compared with the strong signal in the cell lysate and likely reflect minimal co-isolation of soluble proteins during vesicle isolation.

The combined assessment of particle size distribution, vesicular morphology, enrichment of the EV-associated marker CD63, and limited detection of intracellular contaminants (GAPDH and Grp94) supports the successful isolation of EV-enriched preparations from both conditioned medium and mineralized extracellular matrix. However, because only a limited panel of molecular markers was evaluated, these data should be considered supportive rather than a comprehensive molecular characterization according to current MISEV recommendations.

### 2.3. Differential mRNA Cargo Profiling of MEs and MBE-Enriched Fractions

To further verify the cellular origin of the RNA isolated from the EV preparations, rabbit-specific transcripts were assessed in FBS, exosome-depleted FBS, MEs and MBE-enriched fractions. Rabbit 18S and GAPDH transcripts were readily detected in both ME and MBE-enriched fraction preparations, whereas FBS and exosome-depleted FBS showed no amplification or only background-level amplification (Supplementary Figure S2). These findings support that the RNA analyzed in subsequent experiments predominantly originated from rabbit osteoblast-derived EVs.

**Figure 5:**
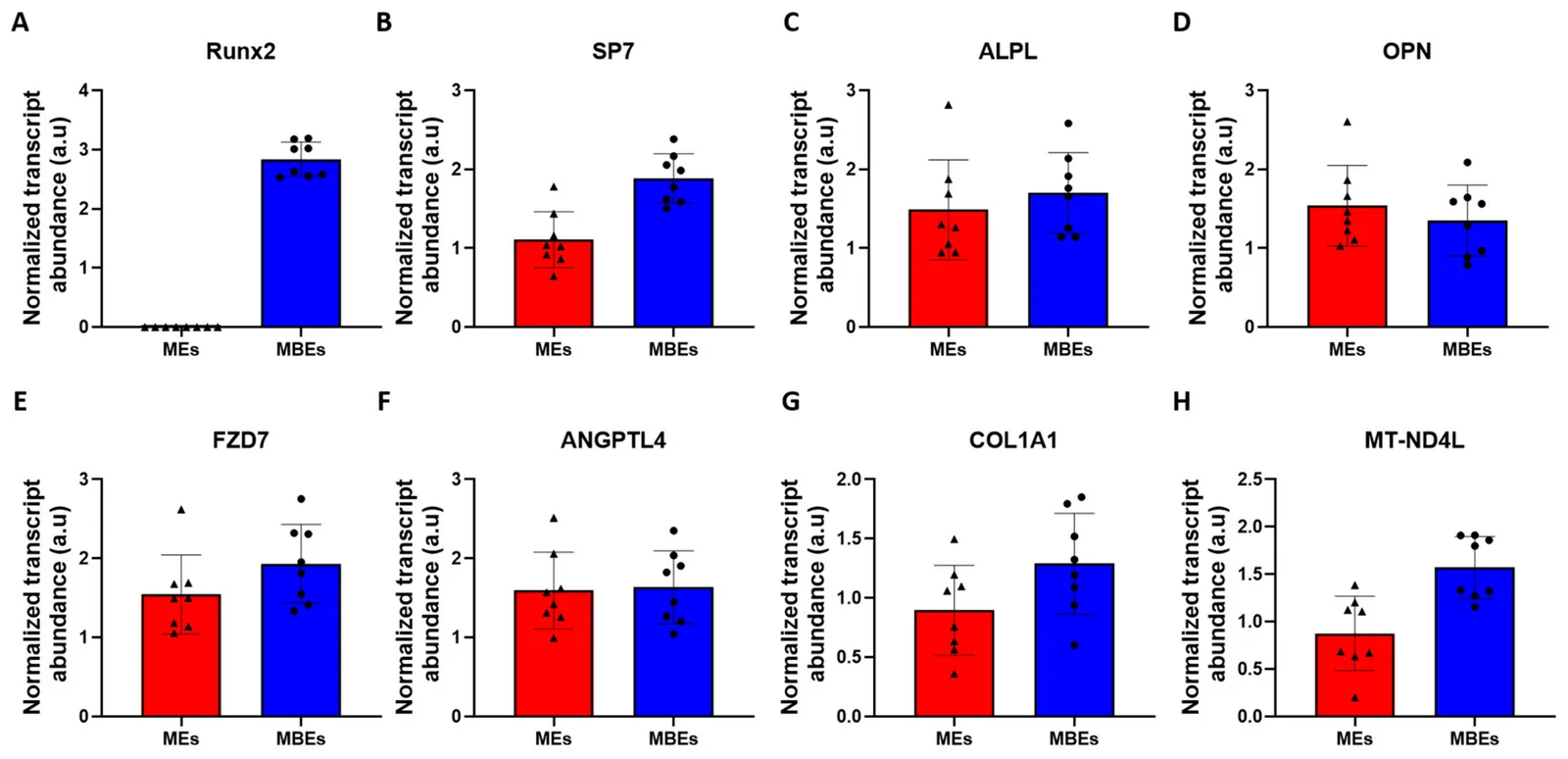
Quantitative analysis of mRNA cargo in media-derived extracellular vesicles (MEs) and matrix-bound extracellular vesicle-enriched fractions (MBEs) isolated from mineralizing osteoblasts. Normalized transcript abundance of (**A**) RUNX2, (**B**) SP7, (**C**) ALPL, (**D**) OPN, (**E**) FZD7, (**F**) ANGPTL4, (**G**) COL1A1, and (**H**) MT-ND4L were assessed by quantitative real-time PCR following RNA extraction from purified vesicle preparations. Data are presented as mean ± SD.

To determine whether MEs and MBE-enriched fractions differed in their nucleic acid cargo despite originating from the same MOB population, we analyzed the expression of selected osteogenesis- and signaling-related mRNAs following RNA extraction from purified vesicle preparations (Figure 5).

Quantitative real-time PCR revealed that most analyzed transcripts, including SP7, ALPL, OPN, FZD7, ANGPTL4, COL1A1, and the mitochondrial transcript MT-ND4L, were detected in both MEs and MBE-enriched fractions at comparable levels, with no statistically significant differences between vesicle populations (Figure 5B–H). In contrast, the osteogenic master transcription factor RUNX2 exhibited a distinct expression pattern. RUNX2 transcripts were robustly detected in MBE-enriched fractions, whereas they were absent or below the detection threshold in ME samples (Figure 5A).

This selective enrichment of RUNX2 mRNA in MBE-enriched fractions indicates differential RNA packaging between vesicle populations and suggests that matrix-associated extracellular vesicles retain molecular features linked to osteogenic commitment. Overall, these data demonstrate that although MEs and MBE-enriched fractions share largely overlapping mRNA profiles, matrix-bound vesicles display a distinct nucleic acid signature characterized by the presence of key osteogenic transcripts.

### 2.4. Effect of MEs and MBE-Enriched Fractions on hBMSCs

#### 2.4.1. Effects of MEs and MBE-Enriched Fractions on mRNA Content of hBMSCs

**Figure 6:**
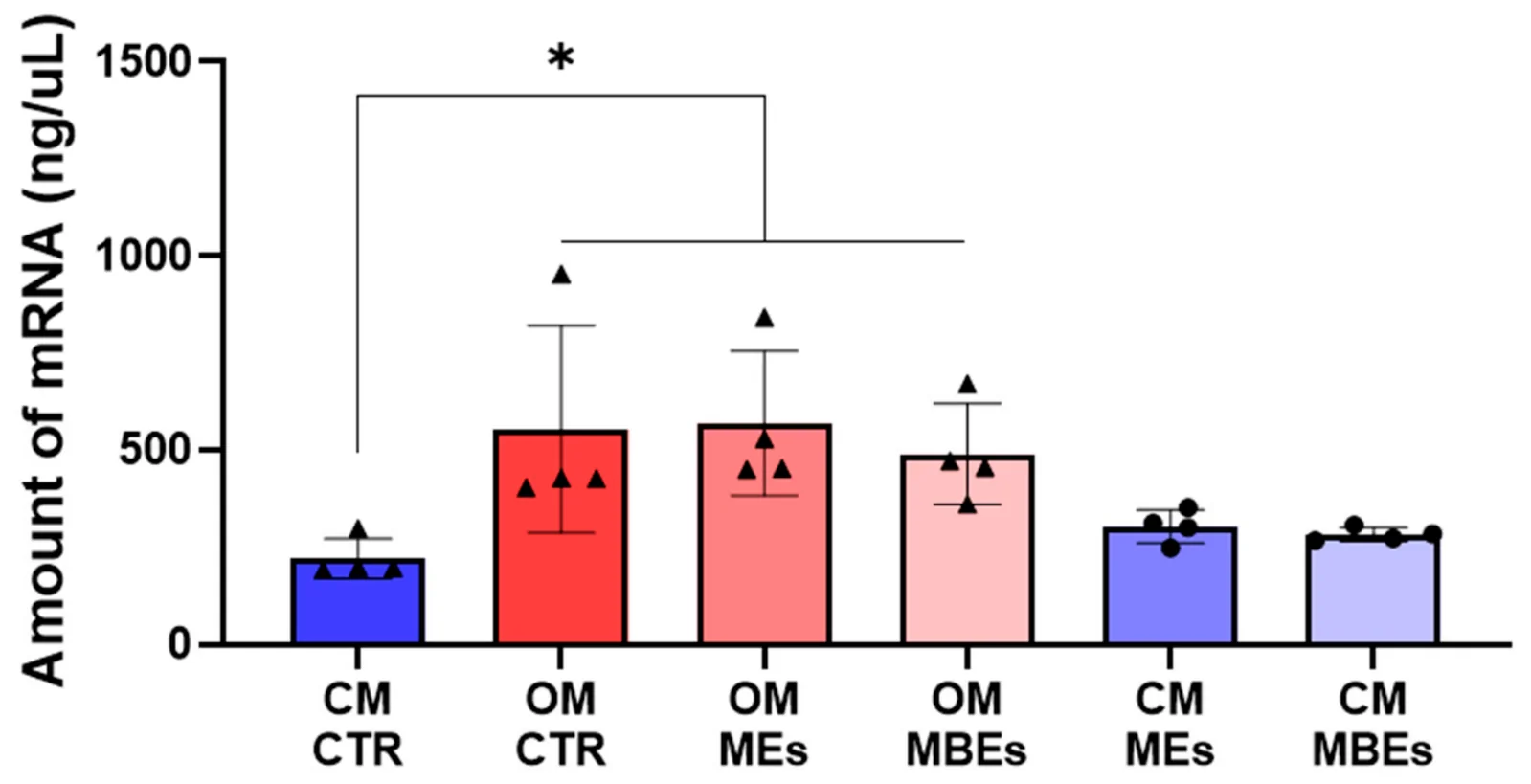
Effects of culture conditions on total RNA yield from hBMSCs after 21 days. RNA amounts were significantly higher in cells cultured in osteogenic medium (OM), with or without extracellular vesicles, compared to the control medium (CM) without extracellular vesicles. Data are presented as mean ± SD. * *p* < 0.05.

On Day 21, RNA was extracted from hBMSC cultures, and total RNA yield was quantified. RNA amounts differed significantly among the experimental conditions (Figure 6). Because RNA content per cell can vary substantially during MSC proliferation and osteogenic differentiation [6], total RNA yield was interpreted as an indicator of global transcriptional activity rather than as a direct measure of cell number.

As shown in Figure 6, after 21 days of culture, hBMSCs maintained in osteogenic medium (OM), in the presence of MEs or MBE-enriched fractions, yielded significantly more RNA compared to cells cultured in the control medium (CM) without EVs. This suggests enhanced transcriptional activity under osteogenic conditions and/or in the presence of EVs.

#### 2.4.2. Effects of MEs and MBE-Enriched Fractions on Osteogenic Gene Expression in hBMSCs

After demonstrating differences in the mRNA content of our EVs, we investigated the effect of MEs and MBE-enriched fractions isolated from MOBs on hBMSCs in a 2D culture model (i.e., cell monolayer). The hBMSCs were cultured for 21 days with EVs added to the culture medium only on Day 1, to closely mimic a clinical setting. The osteogenic differentiation of hBMSCs cultured in 2D was monitored by RT-qPCR, Alkaline Phosphatase, and Alizarin Red staining on Day 21.

**Figure 7:**
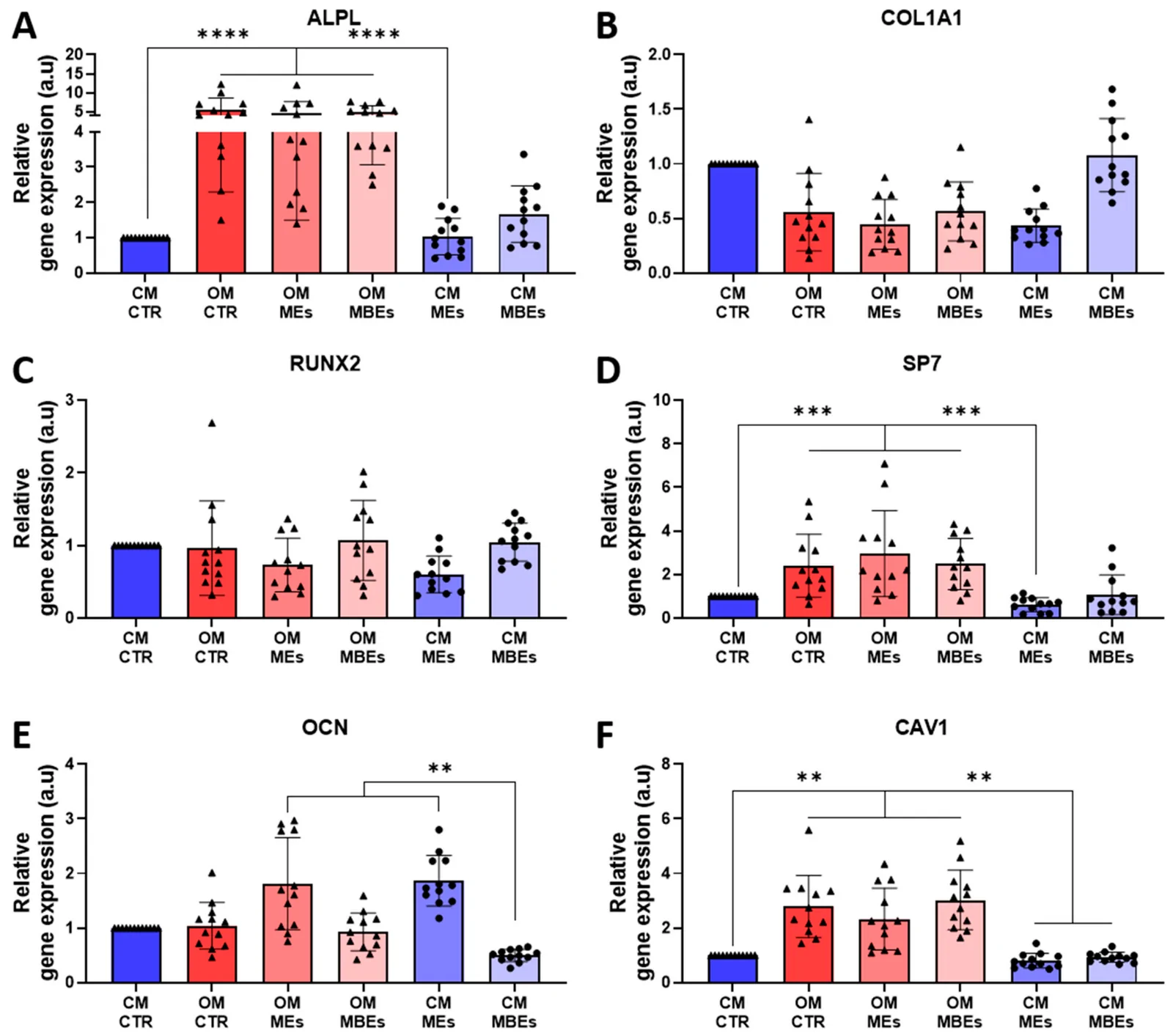
Effects of media-derived extracellular vesicles (MEs) and matrix-bound extracellular vesicle-enriched fractions (MBEs) on osteogenic gene expression in human bone marrow mesenchymal stromal cells (hBMSCs). Relative mRNA expression of osteogenic and mechanotransduction-related genes in hBMSCs after 21 days of culture under control medium (CM) or osteogenic medium (OM) conditions, with or without supplementation with MEs or MBE-enriched fractions derived from mineralizing osteoblasts. (**A**) ALPL, (**B**) COL1A1, (**C**) RUNX2, (**D**) SP7, (**E**) OCN, and (**F**) CAV1. Gene expression levels were quantified by real-time quantitative PCR and normalized to CM control (CM CTR), which was set to 1. Data are presented as mean ± SD. Statistical significance was determined using appropriate parametric or non-parametric tests as described in Section 4. ** *p* < 0.01, *** *p* < 0.001, **** *p* < 0.0001.

To evaluate the osteogenic response of hBMSCs to MEs and MBE-enriched fractions, we quantified the relative mRNA expression of key osteogenic and mechanotransduction-related genes after 21 days of culture under CM or OM conditions (Figure 7). Gene expression levels were normalized to CM control (CM CTR), which was set to 1.

Expression of alkaline phosphatase (ALPL) was significantly increased under all osteogenic conditions (control, with MEs or with MBE-enriched fractions) compared to hBMSCs in culture in CM (control or with MEs) (Figure 7A). Supplementation of OM with MEs or MBE-enriched fractions did not further enhance ALPL expression. In contrast, under CM conditions, neither MEs nor MBE-enriched fractions induced ALPL expression to levels comparable to osteogenic conditions, although a modest increase was observed in hBMSCs culture in CM with MBE-enriched fractions relative to the one culture under CM alone or with MEs.

Analysis of collagen type I alpha 1 chain (COL1A1) expression revealed no significant differences among experimental groups (Figure 7B). COL1A1 transcript levels remained comparable across CM and OM conditions, irrespective of vesicle supplementation. Similarly, expression of the early osteogenic transcription factor RUNX2 showed no statistically significant modulation across the different culture conditions (Figure 7C).

In contrast, SP7 (osterix) expression was significantly upregulated under OM conditions relative to CM CTR (Figure 7D). Both hBMSC cultures under OM with MEs and with MBE-enriched fraction groups displayed elevated SP7 expression compared with CM CTR, whereas vesicle supplementation under CM conditions did not significantly affect SP7 transcript levels.

Expression of the late osteogenic marker osteocalcin (OCN) was significantly increased in ME-treated groups under OM and CM conditions compared to hBMSCs culture under CM with MBE-enriched fractions (Figure 7E), indicating a stronger effect of MEs on late-stage osteogenic differentiation.

Finally, expression of caveolin-1 (CAV1) was significantly upregulated in OM conditions compared with CM CTR (Figure 7F). Both hBMSCs cultured under OM in the presence of MEs or MBE-enriched fractions performed similarly to the OM condition alone for CAV1 expression, with no significant difference between the two vesicle types. Under CM conditions, vesicle supplementation did not significantly alter CAV1 expression.

Collectively, these results indicate that EV supplementation enhances OCN expression in hBMSCs in a differentiation-dependent manner. This effect was observed under osteogenic conditions, with MEs inducing a more pronounced upregulation of the late osteogenic marker osteocalcin compared to MBE-enriched fractions.

#### 2.4.3. Effects of MEs and MBE-Enriched Fractions on ALP Staining

**Figure 8:**
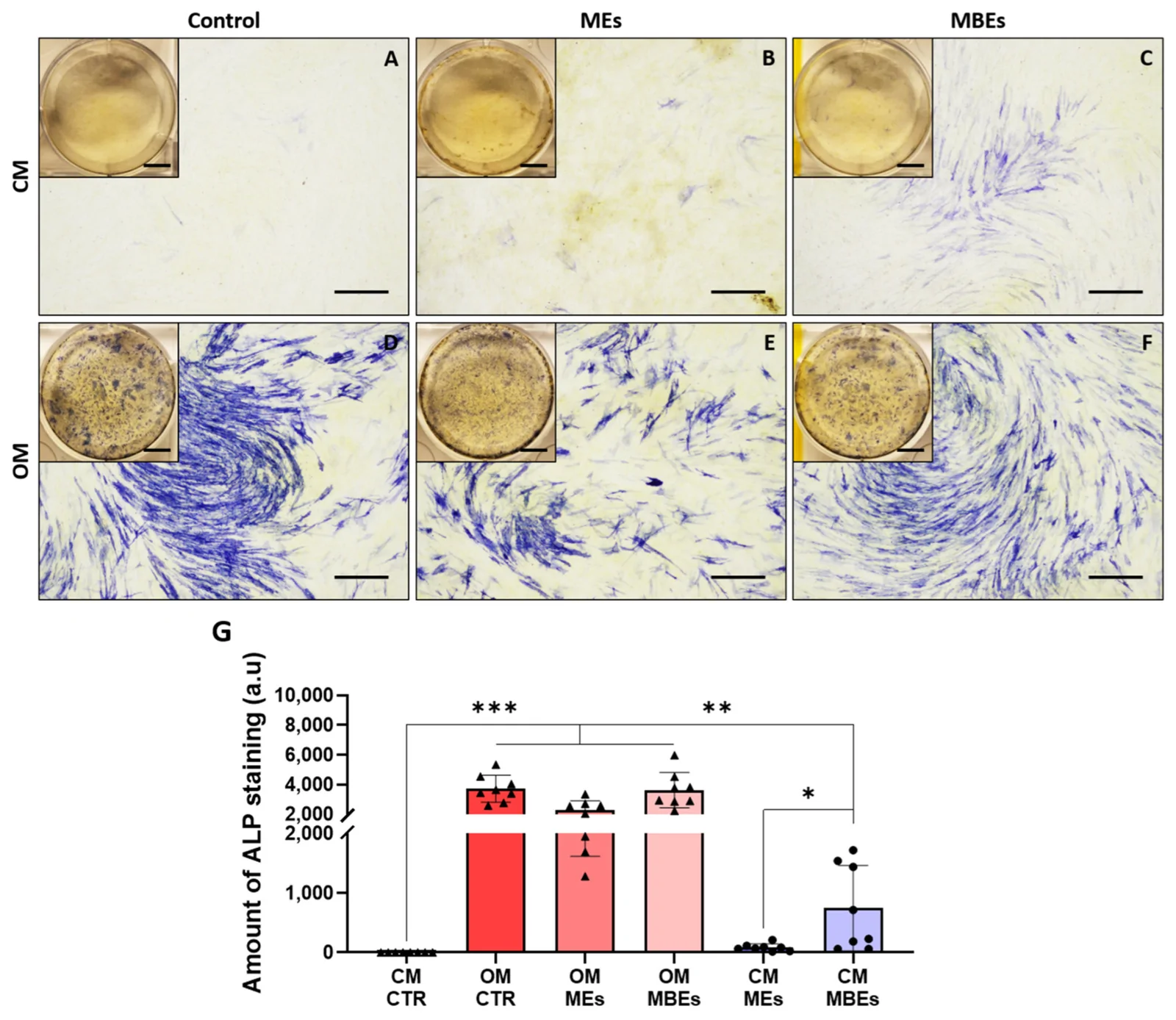
Alkaline phosphatase (ALP) staining and quantification of hBMSCs after 21 days of culture in control medium (CM) or osteogenic medium (OM), with or without media-derived extracellular vesicles (MEs) or matrix-bound extracellular vesicle-enriched fractions (MBEs). Representative images show weak ALP staining in CM (**A**), increased staining in CM supplemented with MEs (**B**) or MBEs (**C**), and intense staining in OM alone (**D**) or OM supplemented with MEs (**E**) or MBE-enriched fractions (**F**). Quantification of ALP-positive staining is shown in (**G**). The scale bar in figures (**A**–**F**) is 200 μm and 5 mm in sub-figures (**A**–**F**). Data are presented as mean ± SD. * *p* < 0.05, ** *p* < 0.01, *** *p* < 0.001.

Given the differences observed in transcriptional activity and osteogenic responses among the experimental conditions, ALP activity was next evaluated as a marker associated with early osteogenic differentiation (Figure 8). In the control condition, hBMSCs cultured in CM showed no detectable ALP staining, as indicated by the absence of purple coloration (Figure 8A). In contrast, positive ALP staining was observed in cells maintained in CM supplemented with MEs (Figure 8B), and with even more stained cells with MBE-enriched fractions (Figure 8C).

At Day 21, hBMSCs cultured in OM exhibited strong ALP staining, both in OM alone and in OM supplemented with MEs or MBE-enriched fractions (Figure 8D–F), indicating robust osteogenic differentiation under these conditions.

Quantitative analysis of ALP staining (Figure 8G) revealed significant differences between hBMSCs cultured in CM and those cultured in OM, irrespective of the presence of MEs or MBE-enriched fractions. Additionally, a significant difference was detected between CM supplemented with MEs and CM supplemented with MBE-enriched fractions, with higher ALP activity observed in the presence of MBE-enriched fractions.

#### 2.4.4. Effects of MEs and MBE-Enriched Fractions on Matrix Mineralization by hBMSCs

**Figure 9:**
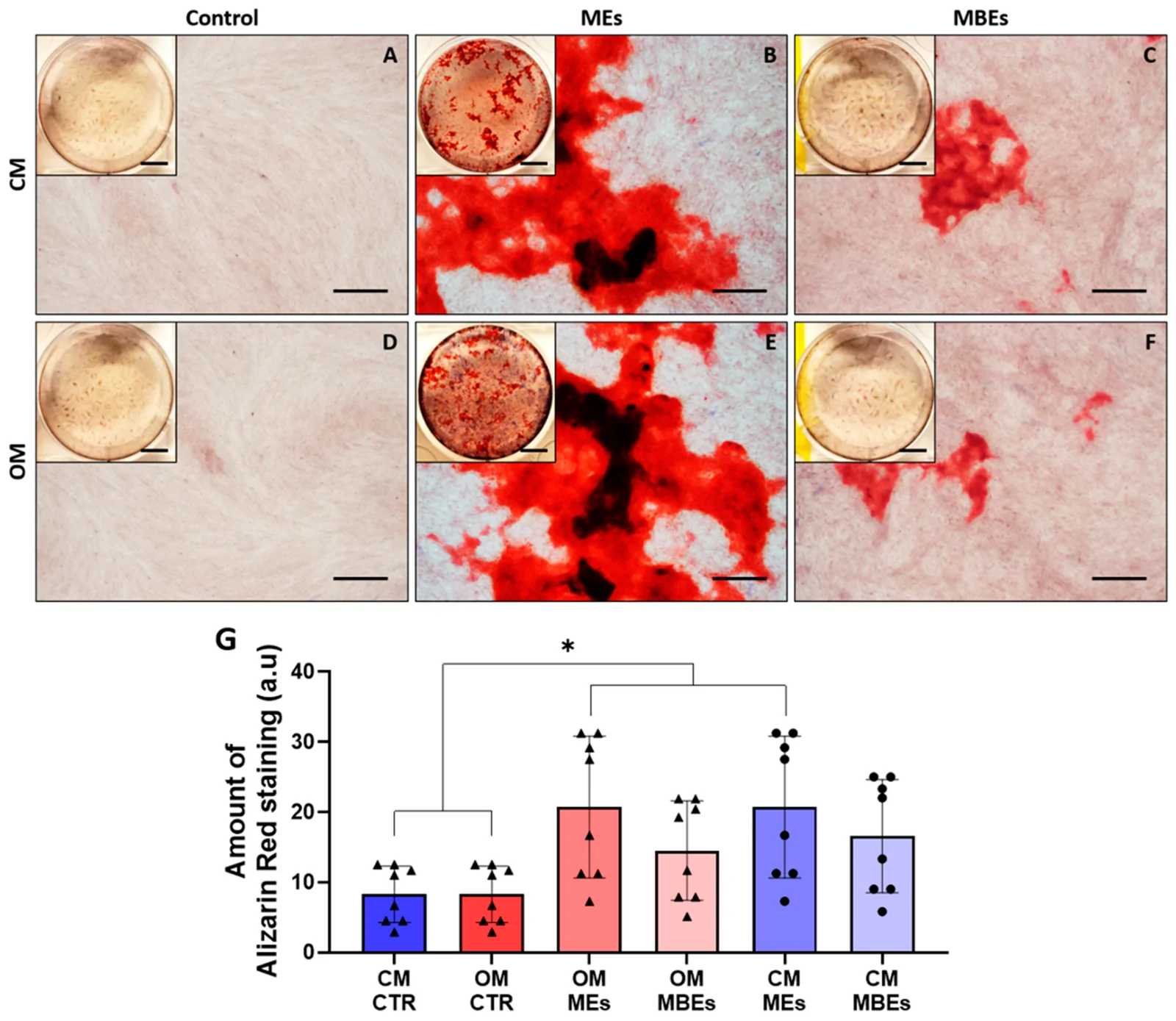
Effects of media-derived extracellular vesicles (MEs) and matrix-bound extracellular vesicle-enriched fractions (MBEs) on matrix mineralization by human bone marrow mesenchymal stromal cells (hBMSCs). Representative Alizarin Red staining images of hBMSCs after 21 days of culture under control medium (CM) or osteogenic medium (OM) conditions, with or without supplementation with MEs or MBE-enriched fractions derived from mineralizing osteoblasts. Panels show (**A**) CM control (CM CTR), (**B**) CM supplemented with MEs (CM MEs), (**C**) CM supplemented with MBE-enriched fractions (CM MBEs), (**D**) OM control (OM CTR), (**E**) OM supplemented with MEs (OM MEs), and (**F**) OM supplemented with MBE-enriched fractions (OM MBEs). Alizarin Red staining highlights calcium-rich mineral deposits indicative of matrix mineralization. Images are representative of at least three independent experiments. Quantification of Alizarin Red staining is shown in (**G**). The scale bar in figures (**A**–**F**) is 200 μm and 5 mm in sub-figures (**A**–**F**). Data are presented as mean ± SD. * *p* < 0.05.

To determine whether the differences in ALP activity were associated with matrix mineralization, Alizarin Red S staining was performed to assess calcium deposition after 21 days of culture. Matrix mineralization by hBMSCs was evaluated under CM or OM conditions, with or without supplementation with MEs or MBE-enriched fractions (Figure 9).

In CM conditions, hBMSCs cultured without EV supplementation showed no detectable Alizarin Red staining, indicating the absence of mineralized matrix deposition (Figure 9A). In contrast, positive Alizarin Red staining, evidenced by red coloration, was observed in hBMSCs cultured in CM supplemented with either MEs (Figure 9B) or MBE-enriched fractions (Figure 9C), with more pronounced staining in the presence of MEs.

Under OM conditions, hBMSCs cultured without EV supplementation exhibited little to no Alizarin Red staining (Figure 9D). However, supplementation of OM with MEs (Figure 9E) or MBE-enriched fractions (Figure 9F) resulted in clear positive Alizarin Red staining, again with more intense staining observed in cultures treated with MEs.

Quantitative analysis of Alizarin Red staining (Figure 9G) revealed significant differences between hBMSCs cultured in the presence of MEs (CM or OM) and those cultured in CM or OM.

Overall, these results indicate that EV supplementation enhances matrix mineralization by hBMSCs under both control and osteogenic conditions. Although Alizarin Red staining was numerically higher in cultures supplemented with MEs than with the MBE-enriched fraction, direct comparisons between the two EV preparations did not reach statistical significance.

## 3. Discussion

In this study, we characterized MEs and MBE-enriched fractions isolated from MOBs and evaluated their functional effects on hBMSCs. Our results demonstrate that, despite similar biophysical characteristics, MEs and MBE-enriched fractions differ in molecular cargo and exert distinct effects on osteogenic differentiation, highlighting functional heterogeneity linked to vesicle origin and microenvironmental context.

Nanoparticle tracking analysis revealed that MEs and MBE-enriched fractions exhibited highly comparable size distributions and concentrations, with the majority of detected particles exhibiting diameters between 30 and 200 nm. However, particle size alone cannot establish EV identity, as NTA does not distinguish vesicular from non-vesicular particles. These findings are consistent with prior reports indicating that EVs isolated from distinct extracellular compartments can share biophysical properties despite divergent biological functions [34,35]. Transmission electron microscopy provided complementary ultrastructural characterization of both EV populations. Consistent with the NTA results, MEs and MBE-enriched fractions exhibited the characteristic rounded morphology of extracellular vesicles, with diameters ranging from approximately 30 to 200 nm. These observations further support that both isolation procedures recovered vesicles within the expected EV size range and are consistent with current recommendations advocating the use of complementary analytical approaches for extracellular vesicle characterization. In addition to confirming vesicle morphology, TEM provides structural evidence that the isolated particles correspond to membrane-bound vesicles rather than exclusively non-vesicular nanoparticles [36]. The slightly smaller diameters measured by TEM likely reflect dehydration and flattening during negative staining, whereas NTA measures the hydrodynamic diameter of particles in suspension. Western blot analyses demonstrated enrichment of the EV-associated marker CD63 together with minimal detection of the intracellular proteins GAPDH and Grp94, supporting the successful isolation of EV-enriched preparations. However, because only a limited number of positive and negative markers were evaluated, this molecular characterization represents a partial implementation of the MISEV recommendations rather than a comprehensive assessment of EV identity [6]. Interestingly, GAPDH was not detected in either the ME or MBE-enriched fraction populations, despite its strong presence in cell lysates. Although GAPDH is frequently reported as a cytosolic EV-associated protein, its detection is not consistent across all EV preparations. According to MISEV2018 and MISEV2023 guidelines, cytosolic proteins such as GAPDH are considered supportive but non-essential markers, and their presence may vary depending on vesicle subtype and isolation methodology. Indeed, proteomic analyses have demonstrated substantial heterogeneity in EV cargo composition, with distinct subpopulations exhibiting differential enrichment of cytosolic proteins [37]. Moreover, recent studies have highlighted that some cytosolic proteins detected in EV preparations may originate from co-isolated non-vesicular components rather than genuine vesicular cargo [38]. Importantly, this observation was consistent across three independent experiments, in which the GAPDH protein was reproducibly detected in cell lysates but remained undetectable in EV fractions. Notably, while the GAPDH protein was not detected in our EV fractions, GAPDH transcripts were detectable at the mRNA level (reference gene), supporting the concept that RNA and protein cargoes are selectively and independently sorted into extracellular vesicles [39,40]. In this context, the absence of detectable GAPDH protein in our preparations likely reflects efficient exclusion of soluble cytosolic contaminants during isolation rather than impaired vesicle integrity. Notably, while Grp94 was strongly detected in cell lysates, it was observed only as faint bands in a subset of EV samples (specifically, in ME donors #3 and #4 and in MBE-enriched fraction donors #1 and #2) indicating effective exclusion of intracellular secretory pathway contaminants and supporting the overall purity of the vesicle preparations. Upon close inspection, these very faint residual signals in the extracellular vesicle fractions are negligible compared with the strong signal in the cell lysate and likely reflect minimal co-isolation of soluble proteins associated with the secretory pathway or extracellular matrix components during vesicle isolation. Together, these observations support the successful enrichment of extracellular vesicles in both ME and MBE-enriched fraction preparations while acknowledging that the presence of residual non-vesicular material cannot be completely excluded.

Despite these similarities, MBE-enriched fractions exhibited selective enrichment of RUNX2 transcripts relative to MEs. RUNX2 is a master transcription factor required for osteoblast commitment and early differentiation, driving expression of key osteogenic genes such as ALPL and OCN [34]. The selective presence of RUNX2 mRNA in MBE-enriched fractions suggests that matrix-bound vesicles may retain differentiation-stage-specific molecular cues reflective of the mineralizing osteoblast phenotype. This aligns with evidence that extracellular matrix-associated vesicles constitute a biologically distinct subpopulation capable of local signal retention and spatial regulation of cell fate [11,18,41].

Supplementation of hBMSC cultures with EVs increased total RNA yield, particularly under OM conditions. Since RNA content per cell varies dynamically during MSC proliferation and differentiation [6], this increase likely reflects enhanced global transcriptional activity rather than differences in cell number, suggesting that EVs can potentiate cellular responsiveness to osteogenic cues.

Analysis of osteogenic gene expression revealed a nuanced effect of EV supplementation. Early markers such as ALPL, RUNX2, and COL1A1 were variably modulated, with ALPL elevated under all osteogenic conditions but not significantly enhanced by EVs above OM alone. Conversely, SP7 (osterix) was significantly upregulated in OM with both MEs and MBE-enriched fractions, supporting the role of EVs in reinforcing osteogenic commitment once differentiation has been initiated. These findings are consistent with previous studies showing that osteoblast-derived EVs can enhance osteogenesis via transfer of molecular regulators, including miRNAs that modulate Wnt/β-catenin signaling [34].

The molecular mechanisms responsible for these osteogenic effects are likely multifactorial. Extracellular vesicles have been shown to transport a diverse repertoire of bioactive cargo, including osteogenesis-related mRNAs, microRNAs, proteins, and lipids that regulate gene expression in recipient cells. Several studies have demonstrated that EV-associated cargo can activate signaling pathways central to bone regeneration, including BMP/SMAD, VEGF, and Wnt/β-catenin signaling, thereby promoting osteogenic differentiation while simultaneously supporting angiogenesis, an essential component of successful bone repair [42,43,44,45]. In particular, engineered EVs enriched in BMP-2 and VEGF-A mRNAs have been shown to induce coupled angiogenic and osteogenic regeneration in critical-size bone defects, highlighting the importance of coordinated signaling between these pathways. Although the molecular cargo responsible for the biological effects observed in the present study was not comprehensively characterized, the selective enrichment of RUNX2 transcripts within the MBE-enriched fraction suggests that differential cargo loading may contribute to the distinct functional profiles of the two EV populations. Future transcriptomic, proteomic, and miRNA analyses will therefore be valuable to identify the signaling molecules responsible for these differences and to determine whether distinct EV subpopulations preferentially regulate specific stages of osteogenic differentiation.

Notably, OCN expression, a marker of late osteoblast maturation and mineral deposition, was significantly increased in ME-treated groups under both CM and OM [35]. This indicates that MEs may particularly influence later stages of osteogenic differentiation, possibly through delivery of pro-mineralization cargo or activation of pathways that enhance matrix deposition.

Functional assays revealed differential effects of MEs and MBE-enriched fractions on osteogenic maturation. Both EV populations enhanced ALP staining under control conditions, indicating stimulation of early differentiation events. MBE-enriched fractions induced significantly higher ALP activity than MEs in CM, consistent with their selective enrichment in RUNX2 mRNA and potential to support early commitment and matrix maturation. This selective enrichment is consistent with previous reports indicating that RNA incorporation into extracellular vesicles is an actively regulated process that does not necessarily reflect the overall transcript abundance of the parent cell [40,46]. Although RUNX2 transcripts were selectively enriched in MBE-enriched fractions, this did not translate into increased RUNX2 expression in recipient hBMSCs after 21 days. This may reflect degradation of transferred transcripts, limited translation efficiency, or the transient nature of early RUNX2 induction, which may have occurred before the final analysis time point.

In contrast, Alizarin Red staining demonstrated a tendency toward greater matrix mineralization in cultures treated with MEs, particularly under osteogenic conditions, which correlated with increased OCN expression. However, direct statistical comparisons between the ME and MBE-enriched fraction groups did not demonstrate a significant difference in mineralization. Therefore, these findings suggest differences in biological activity between the two EV preparations but do not support the conclusion that MEs are unequivocally more effective than the MBE-enriched fraction in promoting matrix mineralization. Conversely, the higher ALP activity observed with the MBE-enriched fraction under control conditions, together with the selective enrichment of RUNX2 transcripts within the vesicle preparation, is consistent with a potential role in promoting osteogenic commitment-related signals. However, because all functional analyses were performed at a single late endpoint (Day 21), these observations do not directly demonstrate a temporal sequence of early versus late differentiation regulation. Therefore, the proposed model whereby matrix-associated vesicles preferentially influence early osteogenic processes while media-derived vesicles promote later maturation should be considered as a working hypothesis requiring validation through future kinetic studies incorporating earlier time points and longitudinal assessment of osteogenic progression. These observations align with previous reports showing that EVs from differentiating osteoblasts deliver miRNAs and proteins that activate canonical osteogenesis pathways such as Wnt/β-catenin, which regulate both early differentiation and mineralization [34,35].

Our findings underscore the biological significance of EV heterogeneity based on origin. Although MEs and MBE-enriched fractions derive from the same MOB population, their distinct cargo profiles and functional effects suggest that EV subpopulations carry specialized messages tailored to specific microenvironmental roles. Matrix-associated vesicles can act as localized signaling reservoirs, whereas diffusible MEs may mediate broader paracrine effects. This intrinsic matrix association may also confer translational advantages by naturally promoting local retention, a characteristic that current biomaterial-based EV delivery strategies seek to reproduce in regenerative medicine. Such compartmentalized extracellular vesicle signaling has been proposed as a key mechanism for spatially coordinated tissue regeneration [18,41]. Interestingly, TEM examination revealed the presence of occasional electron-dense matrix-like fragments exclusively within MBE-enriched fraction preparations, whereas such structures were not observed in MEs. Although TEM alone cannot conclusively identify these structures as extracellular matrix components, their exclusive presence in the MBE-enriched fraction is consistent with the enzymatic recovery of vesicles from a mineralized extracellular matrix. Importantly, the vesicles were generally observed as individual particles rather than remaining physically attached to these matrix-like fragments, suggesting that the collagenase-based isolation procedure efficiently liberated matrix-associated vesicles while preserving their structural integrity. These ultrastructural observations further support the concept that MBE-enriched fractions originate from a distinct extracellular compartment rather than simply representing media-derived vesicles isolated using an alternative protocol [36,47,48].

An important consideration when interpreting these findings is that the two EV populations were isolated over different temporal windows. MEs were collected exclusively from conditioned medium during the final 48 h after replacement with EV-depleted serum, whereas the MBE-enriched fraction represents vesicles that accumulated within the extracellular matrix throughout the entire 21-day mineralization period before enzymatic recovery. Consequently, the molecular composition of the MBE-enriched fraction may reflect cumulative deposition and prolonged matrix retention rather than vesicle secretion during the same period as MEs. Therefore, the functional differences observed between the two preparations should not be attributed solely to their extracellular localization, as temporal differences in vesicle accumulation may also contribute to their distinct molecular cargo and biological activities. While this represents an inherent limitation of current approaches for isolating matrix-bound EVs, future studies employing pulse-labeling techniques, sequential matrix extraction, or alternative time-resolved isolation strategies will be important to distinguish the respective contributions of extracellular compartmentalization and vesicle age to their biological function.

Interestingly, most comparisons between OM alone and OM supplemented with MEs or MBE-enriched fractions did not reach statistical significance. This observation suggests that OM conditions may already induce a near-maximal osteogenic differentiation response in hBMSCs, thereby limiting the capacity to detect additional effects of exogenous EV supplementation. In this context, the absence of significant differences should be interpreted with caution and may reflect a ceiling effect rather than a lack of EV biological activity. Consistent with this interpretation, several studies have reported that extracellular vesicles exert more pronounced functional effects under suboptimal or non-osteogenic conditions, where they can act as initiators or modulators of differentiation pathways rather than further enhancing an already strongly induced phenotype [49,50,51,52]. Therefore, the contribution of MEs and MBE-enriched fractions in the present study may be context-dependent, and their osteogenic potential could be more clearly revealed in less permissive or early-stage differentiation settings.

A potential limitation of this study relates to the contribution of extracellular vesicles present in serum-containing media during earlier stages of culture, which may incorporate into the developing mineralized extracellular matrix. As a result, vesicles isolated from matrix fractions may include a proportion of serum-derived EVs. Although EV-depleted serum was used during the final 48 h prior to isolation to reduce this contribution, it is not possible to exclude the presence of pre-incorporated serum-derived vesicles. Although the contribution of serum-derived EVs cannot be completely excluded, the reproducible molecular and functional differences observed between MEs and MBE-enriched fractions isolated from the same cultures support the conclusion that these populations are biologically distinct. Nevertheless, future studies performed entirely under long-term EV-depleted or chemically defined culture conditions will be required to determine the precise contribution of serum-derived vesicles.

An additional consideration concerns structural characterization of the vesicle populations. In the present study, vesicle identity was assessed using a combination of NTA to determine particle size distribution and concentration, together with Western blot detection of EV-associated markers and evaluation of intracellular contaminants. This multi-parameter approach is in line with the recommendations of the International Society for Extracellular Vesicles (MISEV guidelines), which emphasize the need for complementary analytical methods to characterize EV preparations. Consistent size distributions and protein marker profiles were observed and are in agreement with previous studies from our laboratory describing osteoblast- and MSC-derived vesicles [33,53]. Nevertheless, the present molecular characterization remains limited. Current MISEV guidelines recommend the inclusion of multiple EV-associated markers representing distinct protein categories, together with a broader panel of negative markers to better distinguish extracellular vesicles from co-isolated non-vesicular components. In the present study, only the transmembrane EV marker CD63, together with the negative markers GAPDH and Grp94, was evaluated. Although these markers provide supportive evidence for successful enrichment of extracellular vesicles, additional characterization using markers such as CD9, CD81, Alix, TSG101, syntenin-1, or flotillin-1, together with markers of potential extracellular matrix or protein aggregate contaminants, would further strengthen the molecular characterization of both EV preparations. However, it is important to note that NTA does not discriminate between vesicular particles and non-vesicular entities such as protein aggregates or extracellular matrix fragments [54]. In addition, the use of PEG-based precipitation and matrix digestion steps, particularly for MBE-enriched fraction isolation, may favor the co-isolation of non-vesicular components. Although efforts were made to minimize such contamination, including centrifugation and washing steps, the presence of residual matrix-associated materials cannot be fully excluded and may contribute to the observed biological effects.

Transmission electron microscopy provided morphological evidence consistent with vesicular structures and corroborated the particle size distribution obtained by NTA. Nevertheless, TEM also revealed occasional matrix-like material in MBE-enriched fraction preparations, highlighting the inherent difficulty of completely separating matrix-associated vesicles from their surrounding extracellular environment. Such observations are consistent with the biological origin of matrix-bound vesicles, which are naturally embedded within the extracellular matrix and require enzymatic digestion for their recovery. Future studies employing higher-resolution approaches, including cryo-electron microscopy, electron tomography, or immunogold labeling of EV-associated markers, would further distinguish bona fide extracellular vesicles from co-isolated matrix-derived components and provide deeper insight into matrix–vesicle interactions [55,56].

Finally, the cross-species experimental design should also be considered when interpreting the results. EVs used in this study were derived from rabbit osteoblast cultures and subsequently applied to human bone marrow mesenchymal stromal cells. Although EV-mediated communication is widely conserved across mammalian species and vesicle uptake occurs through common cellular mechanisms such as endocytosis and membrane fusion, species-specific differences in vesicle cargo composition or receptor interactions may influence the magnitude of the observed biological responses. Therefore, while the present results indicate distinct biological profiles between MEs and the MBE-enriched fraction populations, caution should be taken when extrapolating absolute osteogenic effects to fully human systems. Future studies using human osteoblast-derived EVs would further clarify the translational relevance of these findings.

From a translational perspective, the present findings further support the concept that extracellular vesicle subpopulations may be strategically exploited according to their biological properties rather than being considered as a homogeneous therapeutic product. Although the matrix-bound extracellular vesicle-enriched fraction did not demonstrate globally superior osteogenic activity, its natural association with the extracellular matrix may provide important advantages for localized delivery. One of the major challenges limiting the clinical translation of EV-based therapeutics is the rapid diffusion and clearance of vesicles following administration, resulting in limited retention at the target site. Considerable efforts have therefore focused on improving EV potency and local persistence through donor-cell engineering, therapeutic cargo enrichment, and biomaterial-assisted delivery systems [57]. In this context, matrix-associated EVs may represent an attractive biological model for designing scaffold-functionalized regenerative therapies, as their natural retention within the extracellular matrix could facilitate sustained local presentation of bioactive signals during bone repair. Furthermore, advances in parental cell engineering and cargo modulation may enable the generation of EV populations with enhanced osteogenic and angiogenic properties, while biomaterial-based delivery platforms could provide spatial and temporal control over EV release [57]. Nevertheless, successful clinical translation will also require standardized manufacturing protocols, robust quality control, scalable production methods, and a better understanding of the molecular mechanisms responsible for EV bioactivity [58]. Together, these developments may ultimately enable the rational design of next-generation EV-based therapies for bone regeneration [35].

## 4. Materials and Methods

### 4.1. Cell Isolation

#### 4.1.1. Isolation of Rabbit OBs

Rabbit OBs were isolated from calvaria flat bone pieces obtained from healthy adult New Zealand White rabbits. The rabbits were housed and cared for according to approved protocols and guidelines in accordance with the EU Directive 2010/63/EU for animal experiments (65/2018; 90/2021). Calvarial bone pieces were aseptically harvested and meticulously dissected to eliminate connective tissues. The cleaned bone pieces were washed extensively with phosphate-buffered saline (PBS) containing 1% penicillin-streptomycin (PS, #15140122, Gibco, Waltham, MA, USA). The bone pieces were finely minced into fragments of 1–2 mm in size and seeded into tissue culture flasks. These fragments were cultured in CM composed of alpha-minimal essential medium (α-MEM, #12571063, Gibco) supplemented with 10% fetal bovine serum (FBS, #16000044, Gibco), 1% HEPES (#15630080, Gibco), 1% sodium pyruvate (#11360070, Gibco), and 1% penicillin-streptomycin-glutamine (PSG, #10378016, Gibco). The cultures were maintained at 37 °C in a 5% CO_2_ humidified atmosphere. Passage 0 cells were cultured until they reached 70–80% confluency and were then passaged using trypsin-EDTA solution (#25200056, Gibco) for further use, including EV extraction.

#### 4.1.2. Culture of Human BMSCs

Human bone marrow-derived mesenchymal stromal cells (hBMSCs; POIETICS™, #PT-2501, Lonza, Basel, Switzerland) were cultured according to the manufacturer’s instructions. After thawing, cells were seeded into tissue culture flasks and maintained in CM composed of alpha-minimal essential medium (α-MEM, #12571063, Gibco) supplemented with 10% fetal bovine serum (FBS, #16000044, Gibco), 1% HEPES (#15630080, Gibco), 1% sodium pyruvate (#11360070, Gibco), and 1% penicillin-streptomycin-glutamine (PSG, #10378016, Gibco). The cultures were maintained at 37 °C in a 5% CO_2_ humidified atmosphere. Cell adhesion, morphology, and proliferation were monitored routinely. The stromal identity of the cells was confirmed by their capacity for trilineage differentiation (osteogenic, adipogenic, and chondrogenic) as previously described [59]. Passage 1 cells were expanded until reaching 70 to 80% confluence, then subcultured using trypsin-EDTA. Cells at passages 4 to 5 were used for all experiments.

### 4.2. Extracellular Vesicle Isolation and Characterization

#### 4.2.1. Isolation of MEs and MBE-Enriched Fractions from MOBs

Isolation of MEs and MBE-enriched fractions from MOBs was performed using polyethylene glycol (PEG)-based precipitation as previously described [60,61]. Briefly, rabbit osteoblasts at passage 1 were cultured to confluence and then induced toward osteogenic differentiation by replacing the culture medium with OM, consisting of complete α-MEM supplemented with 50 μM ascorbic acid (#A8960, Sigma-Aldrich, St. Louis, MO, USA), 10 mM β-glycerophosphate (#G9422, Sigma-Aldrich), and 100 nM dexamethasone (#D2915, Sigma-Aldrich). Cultures were maintained under these conditions for 21 days to generate mineralized osteoblasts (MOBs).

Following mineralization, OM was replaced with exosome-depleted medium (α-MEM supplemented with 10% exosome-depleted FBS, #A2720801, Gibco, 1% HEPES, 1% sodium pyruvate, and 1% penicillin-streptomycin-glutamine solution). For the isolation of MEs, after 48 h, conditioned media were collected and cleared by sequential centrifugation at 300× *g* for 10 min and 2000× *g* for 30 min to remove cells and debris. The replacement with EV-depleted serum during the final 48 h was intended to minimize contamination by newly introduced serum-derived extracellular vesicles before isolation. Concerning the isolation of MBE-enriched fractions, mineralized ECM was digested using 2 mg/mL collagenase type II (#LS004176, Worthington Biochemical Corporation, Lakewood, NJ, USA) for 1 h at 37 °C. The resulting ECM digest was centrifuged at 2500× *g* for 30 min to remove cellular remnants and debris.

For both ME- and MBE-containing supernatants, PEG precipitation was carried out by adding 16% PEG6000 (#81260, Sigma-Aldrich) containing 1 M NaCl (#71376, Sigma-Aldrich) at a 1:1 (*v*/*v*) ratio, followed by overnight incubation at 4 °C. Samples were centrifuged at 2500× *g* for 30 min at 4 °C, and the resulting EV pellets were resuspended in PBS. EV size distribution and particle concentration were quantified using nanoparticle tracking analysis (NTA) on a NanoSight instrument (Malvern, version 3.1, Worcestershire, UK) as previously described [33].

#### 4.2.2. Characterization of MEs and MBE-Enriched Fractions from MOBs

##### Nanoparticle Tracking Analysis (NTA)

The size distribution and concentration of EVs were measured using NanoSight NS300 (Malvern Panalytical, Malvern, UK). EVs were diluted in PBS to achieve a concentration of 25–75 particles/frame. The solution was inserted into a 488 nm laser chamber at a constant rate, and five videos with a duration of 60 s were recorded. NTA software (NTA 3.1 Build 3.1.54, Malvern Panalytical, Malvern, UK) analyzed the data with a detection threshold of 5 and a camera level of 15. For EV concentration calculation, only particles in the range of 30 to 200 nm were considered.

##### Negative Staining for Transmission Electron Microscopy (TEM)

Freshly isolated EVs (MEs and MBE-enriched fractions) were characterized by negative-staining transmission electron microscopy (TEM) to assess their morphology and size. Carbon-coated 300-mesh copper grids (EMS CF300-CU-50; Electron Microscopy Sciences, Hatfield, PA, USA), pre-coated with a 5–10 nm continuous carbon film, were glow-discharged immediately before use. A 3 to 5 µL aliquot of freshly isolated EV suspension was deposited onto the carbon-coated side of the grid and allowed to adsorb for 60 s. Excess liquid was gently removed by blotting from the edge of the grid with filter paper while maintaining the carbon film moist. The specimen was immediately negatively stained with 1% (*w*/*v*) uranyl acetate (freshly prepared, 0.2 µm filtered) for 60 s, followed by gentle blotting and air drying.

Grids were imaged using an FEI Talos 120 transmission electron microscope (Thermo Fisher Scientific, Waltham, MA, USA) operated at an accelerating voltage of 120 kV and equipped with a LaB_6_ electron source and a bottom-mounted BM-Ceta 4 k × 4 k CMOS camera. Representative micrographs were acquired under automated imaging conditions using MAPS 3 software (Thermo Fisher Scientific). ImageJ 1.54s (National Institutes of Health, Bethesda, MD, USA) was used to calculate the diameter of isolated particles.

##### Western Blot

Total cell lysates of the OBs, MOBs and extracellular vesicle proteins isolated from them were extracted using RIPA buffer supplemented with protease and phosphatase inhibitors. The samples were loaded on a 4–20% precast polyacrylamide gel to start the electrophoresis and the gels were transferred to a PVDF membrane using the precast Trans-Blot turbo stack (BioRad, Hercules, CA, USA). Blocked with 5% non-fat milk in TBS (Tris-buffered saline) containing 0.1% Tween-20, membranes were incubated with primary antibodies against CD63 (#ab217345, Abcam, at 1:1000, Cambridge, UK), GAPDH (#sc-32233; Santa Cruz Biotechnology, Dallas, TX, USA, at 1:1000) or Grp94 (#7184-RTM1X-P1; SPM249, Thermo Fischer Scientific, Waltham, WA, USA, at 1:1000). An anti-mouse IgG HRP (horse radish peroxidase)-linked antibody (#7076, Cell Signaling Technology, Danvers, MA, USA, at 1:2000) or anti-rat IgG HRP-linked antibody (#7077S, Cell Signaling Technology, at 1:2000) was used as the secondary antibody. The signals of the proteins were detected using Clarity Western ECL (Hercules, CA, USA, Enhanced Chemiluminescence) substrate in conjunction with the ChemiDoc MP imaging system (BioRad).

### 4.3. 2D Cell Culture of hBMSCs with MEs and MBE-Enriched Fractions from MOBs

To examine the impact of MEs and MBE-enriched fractions from MOBs on human stromal cells, hBMSCs were seeded at 10,000 cells/cm^2^ in six-well culture plates. After 4 h, the culture medium (2 mL per well) was supplemented or not with osteogenic growth factors and MEs or MBE-enriched fractions from MOBs. Each preparation was administered at a standardized particle-based dose of 5.0 × 10^8^ particles per well, corresponding to a final concentration of 2.5 × 10^8^ particles/mL, diluted in 50 µL of PBS. After 21 days of culture, hBMSC cultures supplemented with EVs on Day 1 were harvested and analyzed using PCR and histological staining.

### 4.4. RNA Extraction and Real-Time PCR

Total RNA was extracted either from isolated MEs and MBE-enriched fractions (corresponding to 1.0 × 10^9^ particles) or from hBMSC monolayers after 21 days of culture. Samples were lysed in 1000 µL of QIAzol Lysis Reagent (#79306, Qiagen, Hilden, Germany), and RNA extraction was performed using the RNeasy Mini Kit (#74004, Qiagen) according to established protocols [33]. Briefly, 200 μL chloroform was added, and samples were vortexed for 15 s and centrifuged at 10,000× *g* for 15 min at 4 °C. The aqueous phase (~700 μL) was collected, mixed with 700 μL of 100% ethanol, and purified using RNeasy spin columns following the manufacturer’s instructions, including on-column DNase digestion with RNase-free DNase (#79254, Qiagen). RNA was eluted in 50 μL RNase-free water (#129112, Qiagen). RNA quantity and purity were assessed using a NanoDrop 2000 spectrophotometer (Thermo Fisher Scientific).

For cDNA synthesis, 1 µg of total RNA was reverse-transcribed using iScript™ Reverse Transcription Supermix (#1708840, Bio-Rad) according to the manufacturer’s protocol. Quantitative PCR was performed in duplicate using a CFX96 Real-Time PCR System (Bio-Rad) and SYBR^®^ Green Supermix (#1708880, Bio-Rad) with gene-specific primers (Bio-Rad; Table 1). Gene expression levels in hBMSCs were normalized to ribosomal protein S18 (18S) and glyceraldehyde-3-phosphate dehydrogenase (GAPDH) using the comparative 2^−ΔΔCt^ method [33]. For the analysis of mRNA cargo in extracellular vesicles, transcript abundance was calculated using the 2^−ΔCt^ method, normalized to the reference genes.

### 4.5. Histological Analysis

Histological analyses of samples were conducted after 21 days of culture by Alkaline phosphatase and Alizarin Red Staining. Briefly, for ALP staining, after 21 days, cells were washed in PBS and fixed with PFA 4% at 4 °C for 15 min. Following fixation, the cells were washed with PBS and then incubated for 20 min at room temperature in a staining solution containing 0.1 mg/mL Naphthol AS-MX phosphate (#N4875), 0.5% N,N-dimethylformamide (aka. DMF) (#D4551), 2 mM MgCl_2_ (#63068), and 0.6 mg/mL Fast Blue BB salt (#F3378) prepared in 0.1 M Tris-HCl buffer (#10812846001), pH 8.5.

Concerning the Alizarin Red staining, it was performed to evaluate the in vitro osteogenic differentiation of hBMSCs at 21 days, following previously described protocols [62]. Briefly, wells were washed with PBS, and cells were fixed with 4% formalin for 15 min at 4 °C. After fixation, hBMSCs were rinsed twice with distilled water, and a 40 mM Alizarin Red solution was added. The cells were incubated for 30 min at room temperature with constant shaking, followed by four washes with distilled water to remove excess stain. Finally, wells were covered with PBS, and the photos were taken using the CKX53 optical microscope (Olympus, Tokyo, Japan). For mineralization quantification, all values were normalized to the control group at each time point (set to 1) to facilitate relative comparisons across experimental conditions. This approach ensures standardized data interpretation while preserving the observed trends in mineral deposition.

### 4.6. Statistical Analysis

Results were expressed as mean ± SD and subjected to statistical analysis following the Shapiro–Wilk test for normal distribution. Non-parametric Kruskal–Wallis or Mann–Whitney tests were applied for data sets that did not meet normality criteria, while parametric 1-way ANOVA or *t*-tests were utilized for data sets demonstrating normality. For multiple-group comparisons, one-way ANOVA with appropriate post hoc multiple-comparison testing, or Kruskal–Wallis testing with appropriate post hoc testing, was used as applicable. Statistical significance was considered at *p* values < 0.05 (* *p* < 0.05, ** *p* < 0.01, *** *p* < 0.001, and **** *p* < 0.0001). All statistical analyses were performed using GraphPad Prism 9 software (GraphPad Software, San Diego, CA, USA).

## 5. Conclusions

In conclusion, this study demonstrates that EVs isolated from the conditioned medium and extracellular matrix of mineralizing OBs exhibit distinct biological activities despite comparable biophysical characteristics. While the MBE-enriched fraction was associated with higher alkaline phosphatase activity and selective enrichment of RUNX2 mRNA, MEs promoted greater osteocalcin expression and a tendency toward matrix mineralization. These findings indicate that EVs originating from different extracellular compartments may differentially influence osteogenic differentiation rather than one population displaying uniformly superior osteogenic activity. Because the matrix-bound preparation may also contain extracellular matrix-associated components co-isolated during the extraction procedure, additional studies using more refined purification strategies and expanded EV characterization will be required to further define the specific contribution of matrix-bound EVs. Nevertheless, the present work highlights the importance of considering extracellular compartmentalization when developing EV-based approaches for bone regeneration and tissue engineering.

## Figures and Tables

**Table 1:** List of primers used in this study (for rabbit and/or human samples), with their short name, full name, Ensembl number, NCBI Entrez Gene number, and BioRad unique assay ID.

Short Name	Full Name	Ensembl Number; NCBI Entrez Gene Number (for Rabbit and/or Human)	BioRad Unique Assay ID (for Rabbit and/or Human)
GAPDH	Glyceraldehyde-3-Phosphate Dehydrogenase	ENSOCUG00000025023; 100009074 ENSG00000111640; 2597	qOcuCED0019227 qHsaCED0038674
18S	Ribosomal Protein S18	ENSOCUG00000025023; 100009074 ENSG00000231500; 6222	qOcuCED0018100 qHsaCED0037454
ALPL	Alkaline Phosphatase, Biomineralization Associated	ENSOCUG00000027644; 100347991 ENSG00000162551; 249	qOcuCED0012587 qHsaCED0045991
COL1A1	Collagen Type I Alpha 1 Chain	ENSOCUG00000004447; 100341109 ENSG00000108821; 1277	qOcuCED0015663 qHsaCED0043248
RUNX2	RUNX Family Transcription Factor 2	ENSOCUG00000012881; 100347598 ENSG00000124813; 860	qOcuCID0006259 qHsaCID0006726
SP7	Osterix, Sp7 Transcription Factor	ENSOCUG00000022536; 100008982 ENSG00000170374; 121340	qOcuCED0015349 qHsaCED0003759
OPN	Osteopontin, Secreted Phosphoprotein 1	ENSOCUG00000022536; 100008982	qOcuCED0010864
OCN	Osteocalcin	ENSG00000242252; 632	qHsaCED0038437
CAV1	Caveolin 1	ENSG00000105974; 857	qHsaCID0022177
FZD7	Frizzled Class Receptor 7	ENSOCUG00000011457; 103348917	qOcuCED0015492
ANGPTL4	Angiopoietin-Like 4	ENSOCUG00000008499; 100342802	qOcuCED0008856
MT-ND4L	Mitochondrially Encoded NADH:Ubiquinone Oxidoreductase Core Subunit 4L	ENSOCUG00000016603; 100339191	qOcuCED0019933

## Data Availability

The original contributions presented in this study are included in the article/Supplementary Materials. Further inquiries can be directed to the corresponding author.

## Supplementary Materials

The following supporting information can be downloaded at: https://www.mdpi.com/article/10.3390/ijms27167400/s1.

## Abbreviations

The following abbreviations are used in this manuscript:

ALP	Alkaline Phosphatase
ALPL	Alkaline Phosphatase, Biomineralized Associated
ANGPTL4	Angiopoietin-Like 4
BMP-2	Bone Morphogenetic Protein 2
BMSCs	Bone Marrow Mesenchymal Stromal Cells
CAV1	Caveolin 1
CD63	Cluster of Differentiation 63
CM	Control Medium
COL1A1	Collagen Type I Alpha 1 Chain
CTR	Control
DMF	N,N-Dimethylformamide
ECM	Extracellular Matrix
ECL	Enhanced Chemiluminescence
EDTA	Ethylenediaminetetraacetic Acid
ENS	Ensembl Gene Identifier
EVs	Extracellular Vesicles
FBS	Fetal Bovine Serum
FZD7	Frizzled Class Receptor 7
GAPDH	Glyceraldehyde-3-Phosphate Dehydrogenase
GPI	Glycosylphosphatidylinositol
Grp94	Glucose-Regulated Protein 94
hBMSCs	Human Bone Marrow-Derived Mesenchymal Stromal Cells
HEPES	4-(2-Hydroxyethyl)-1-Piperazineethanesulfonic Acid
HRP	Horseradish Peroxidase
IgG	Immunoglobulin G
MBEs	Matrix-Bound Extracellular Vesicles
MBVs	Matrix-Bound Vesicles
MEs	Media-Derived Extracellular Vesicles
MISEV	Minimal Information for Studies of Extracellular Vesicles
MOBs	Mineralized Osteoblasts
MSCs	Mesenchymal Stromal Cells
MT-ND4L	Mitochondrially Encoded NADH:Ubiquinone Oxidoreductase Core Subunit 4L
MVB	Multivesicular Body
NCBI	National Center for Biotechnology Information
OCN	Osteocalcin
OM	Osteogenic Medium
OPN	Osteopontin
PBS	Phosphate-Buffered Saline
PEG	Polyethylene Glycol
PFA	Paraformaldehyde
PSG	Penicillin-Streptomycin-Glutamine
PVDF	Polyvinylidene Fluoride
RUNX2	RUNX Family Transcription Factor 2
SOX2	SRY-Box Transcription Factor 2
SP7	Sp7 Transcription Factor (Osterix)
TBS	Tris-Buffered Saline
TEM	Transmission Electron Microscopy
TNAP	Tissue-Nonspecific Alkaline Phosphatase
Tris-HCl	Tris(hydroxymethyl)aminomethane Hydrochloride
