# Supplementary material for: Matrix-Bound and Media-Derived Extracellular Vesicles from Mineralized Osteoblasts Exhibit Distinct Osteogenic Activities
Source: Int J Mol Sci. 2026 Aug 19;27(16):7400. doi: 10.3390/ijms27167400 (PMC13513129; doi:10.3390/ijms27167400)
Supplement: Supplementary file 1 [file ijms-27-07400-s001.zip › ijms-4487029-supplementary.pdf]

Supplementary Figure S1

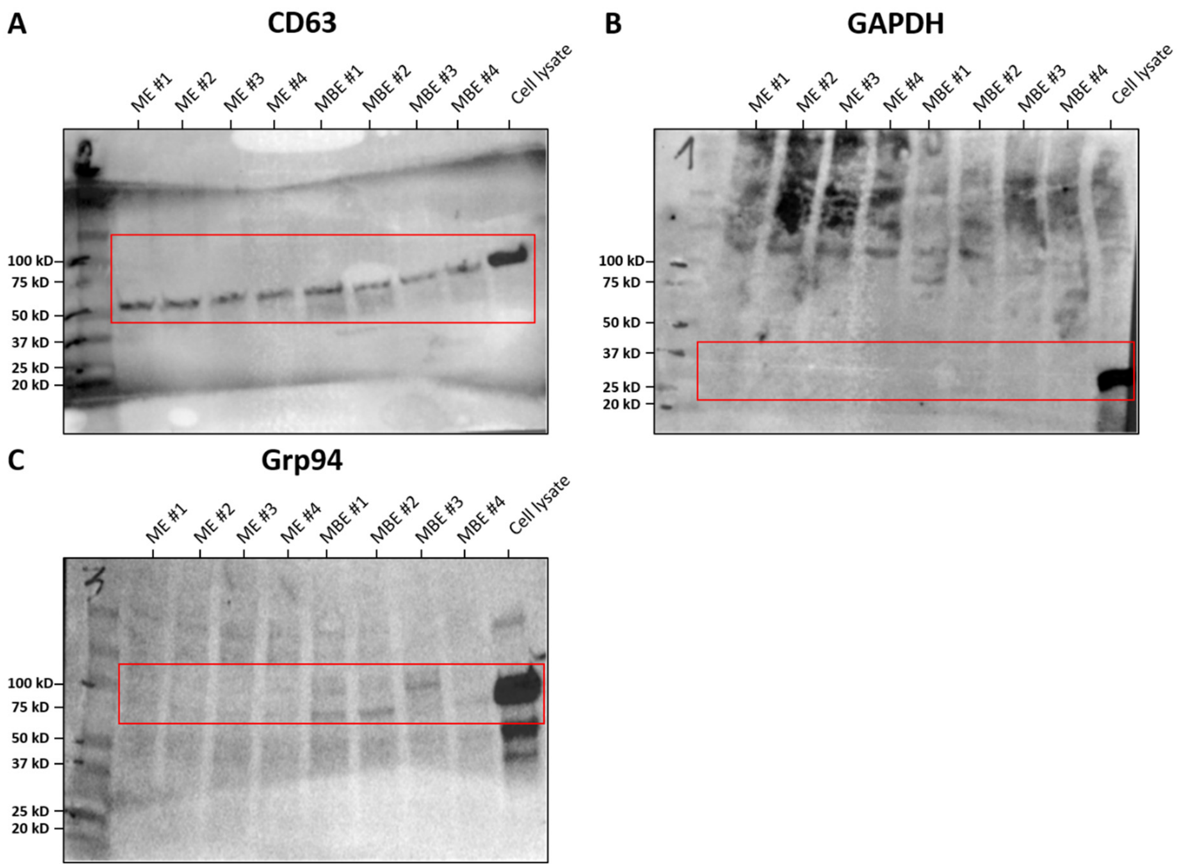

Supplementary Figure S1. Original uncropped Western blot membranes corresponding to Figure 2. Full-length immunoblots showing detection of CD63 (A), GAPDH (B), and Grp94 (C) in media-derived extracellular vesicles (MEs), matrix-bound extracellular vesicles-enriched fraction (MBEs), and cell lysates. Cropped regions used in Figure 2 are indicated by red boxes.

Supplementary Figure S2

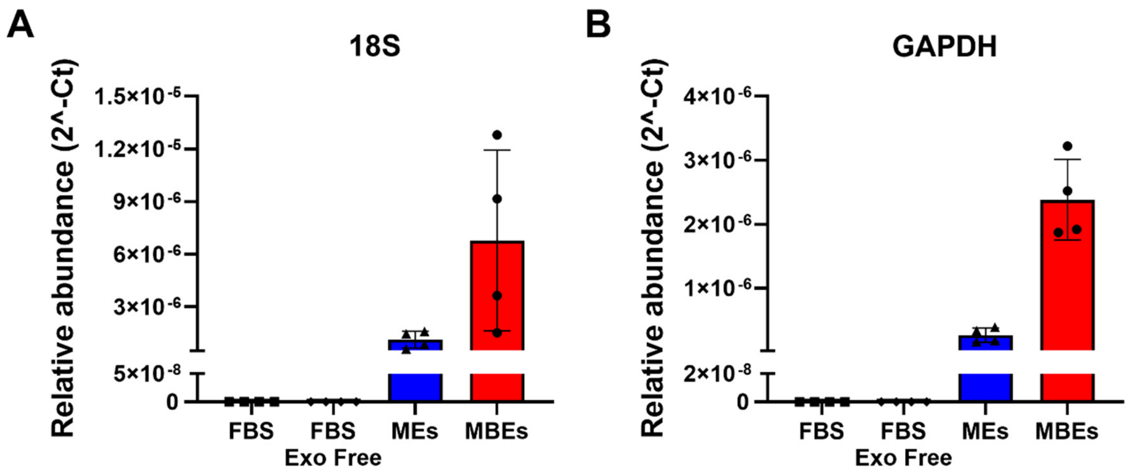

Supplementary Figure S2. Detection of rabbit-specific transcripts in fetal bovine serum and extracellular vesicle preparations.

Rabbit-specific (A) 18S, and (B) GAPDH transcripts were analyzed by RT-qPCR in extracellular vesicles isolated from fetal bovine serum (FBS), exosome-depleted fetal bovine serum (Exo-free FBS), media-derived extracellular vesicles (MEs), and matrix-bound extracellular vesicles-enriched fraction (MBEs). Transcript abundance is presented as 2^-Ct. Rabbit transcripts were readily detected in both ME and MBE preparations, whereas FBS and exosome-depleted FBS exhibited no or only background-level amplification, confirming that the isolated vesicle preparations are enriched in rabbit-derived RNA.
